# Management of Pain and Pruritus in Pediatric Recessive Dystrophic Epidermolysis Bullosa

**DOI:** 10.1002/ccr3.73135

**Published:** 2026-07-10

**Authors:** Maiju K. Marttinen, Reetta Sipilä, Hanna Vuorimaa, Minna Ståhl, Katariina Hannula‐Jouppi

**Affiliations:** ^1^ Päijät‐Häme Wellbeing Services County Lahti Finland; ^2^ Department of Public Health University of Helsinki Helsinki Finland; ^3^ The Finnish Center for Pediatric and Adolescent Pain Management and Research, HUS New Children's Hospital Helsinki Finland; ^4^ SleepWell Research Programme, Faculty of Medicine University of Helsinki Helsinki Finland; ^5^ Department of Dermatology and Allergology, ERN‐Skin Center University of Helsinki and Helsinki University Central Hospital Helsinki Finland; ^6^ Folkhälsan Research Center Helsinki Finland; ^7^ Research Programs Unit, Stem Cells and Metabolism Research Program University of Helsinki Helsinki Finland

**Keywords:** dermatology, pain, pediatric, recessive dystrophic epidermolysis bullosa

## Abstract

The current report presents a tailored and efficient pain management strategy for a 7‐year old boy with recessive dystrophic epidermolysis bullosa severe generalized. Successful management of pain associated with demanding wound care was achieved through a combination of oral pregabalin, topical gabapentin–lidocaine, and psychological interventions.

## Key Clinical Message

Children with recessive dystrophic epidermolysis bullosa *severe generalized* (RDEB‐*SG*) are predisposed to daily pain. Herein, peroral pregabalin showed efficacy for baseline pain, topical gabapentin for itch, and lidocaine gel for wounds. Psychological interventions to improve self‐efficacy and decrease fear of pain play a central role in optimized pain treatment.

## 1 Introduction

Recessive dystrophic epidermolysis bullosa (EB) *severe generalized* (RDEB‐*SG*) is the most severe form of EB, with generalized skin manifestations and, for example, oral, esophageal and anal mucosal lesions, strictures, pseudosyndactyly, limb contractures, nutritional deficiencies and over 90% lifetime risk for aggressive squamous cell carcinoma [1, 2]. Recurrent infections of skin lesions comprise a major problem, and severe infections are not a rarity [1, 3].

RDEB‐*SG* causes substantial physical, emotional, and social suffering for affected individuals [4]. Regular wound care is essential, yet often very painful for pediatric patients. Pain may also be due to moving with contractured limbs and from painful peripheral neuropathy [5]. Adequate management of pain and other disease manifestations is essential for improving quality of life and facilitating necessary wound care in patients with RDEB‐*SG*.

The current report presents a pharmacological pain and pruritus management plan for a pediatric patient with RDEB‐*SG*. The goal was to achieve a favorable balance between efficacy and adverse effects in order to enable adequate wound care, support school attendance, and improve daily functioning and well‐being.

## 2 Case History/Examination

The current paper presents a 7‐year‐old boy with RDEB‐*SG* and no other diagnosed primary illnesses. At the age of 6 months, genome sequencing revealed two heterozygotic mutations in the collagen 7A1 gene (*COL7A1*): c.41 T>A (p.(Ile14Asn)) and c.58C>T (p.(Arg20*)). The parents were heterozygous carriers of the mutations. The patient expressed multiple severe manifestations of RDEB‐*SG*, which are presented in Table 1.

**TABLE 1:** Manifestations of recessive dystrophic epidermolysis bullosa in a 7‐year‐old boy before intensive pain management strategy planning.

	Findings	Symptoms	Complications
Skin	Multiple lesions, wounds, blisters scars and milia on all limbs, trunk and face	Pain, itch, burn, sting	Continuous skin infections
Oral cavity	Slight redness	Occasional pain when swallowing, occasionally refuses to eat	Lloss of weight
Esophagus	N/A	Occasional pain when swallowing	Loss of weight
Anus	Redness, irritation, blisters, wounds	Occasional pain, tries to avoid defecation	Constipation
Fingers	Incipient pseudosyndactyly in both hands (multiple finger gaps risen to PIP joint level), thumbs converging to the center of palms, approximately 30° loss of active total extension in DIP joints.	N/A	N/A
Large joints	Incipient active knee extension loss due to pain	N/A	N/A
Eyes	Slight corneal scarring under the optical axis	N/A	N/A
Growth and nutrition	Weight: 17.2 kg (−27%) Height: 122.4 cm (−1.5 SD) BMI: 48 kg/m^2^ hypoalbuminemia	N/A	Loss of body weight

Abbreviation: BMI, body mass index.

Due to pruritus, pain, a burning sensation, and fear of discomfort, the patient had opposed wound care and showering, which had led to the vicious cycle of continuous infections needing peroral antibiotics. He was on constant paracetamol (15 mg/kg × 3 per os) for pain, yet received minimal subjective benefit. All other pain medications had been discontinued due to lack of efficacy. Hydroxyzine hydrochloride and cetirizine had been tried for pruritus without benefit. Several topical corticosteroid creams and antibacterial agents were used. Emollients were a mainstay of his skin treatment.

The patient expressed fear and avoidance behavior, especially in situations he expected to be painful, for example, showering. Physical restrictions and inability to attend normal activities caused frustration. However, according to a psychologist's evaluation, there were no signs of clinical depression or severe anxiety.

The patient's primary pain type was nociceptive. He also had burning and stabbing sensations with clinical findings of multi‐site allodynia and hyperalgesia, suggesting additional neuropathic pain and likely central sensitization. Pruritus resulted in constant scratching.

The patient did not express continuous baseline pain (pain when resting). However, to weaken overall pain signaling, to enable daily skin treatment, and to possibly prove efficacious for the continuous pruritus [6], pregabalin (1.7 mg/kg × 2 per os) was considered indicated and thus initiated. After 3 months of pregabalin treatment, the patient's mother reported *“*markedly reduced overall pain during activities, yet, he still opposes wound care and especially showering due to pain.” No objective measure for benefit estimation of pregabalin was applicable. No adverse effects were noticed or reported. Skin infections had improved moderately. However, antibiotics were still frequently needed for infected wounds.

## 3 Differential Diagnosis, Investigations and Treatment

After a multidisciplinary Pediatric Pain Center team discussion, accompanied with a dermatologist, plastic surgeon, and the patient's caregivers, a topical analgesic and anesthetics testing day was arranged at a ward in the New Children's Hospital in Helsinki in an attempt to reduce the patient's experience of pain during skin and wound care. The aims of the testing day were:

- To test tolerability of several *ex tempore* topical analgesics and/or anesthetic products.
- To identify products' potential efficacy for different wound types, skin areas, and symptoms.
- To try to improve the patient's self‐efficacy and pain‐related fear and avoidance behavior in wound care situations.

The patient was informed about the purpose of the day and possible (known) adverse effects and sensations. The testing took place in a regular patient room on the ward. He was let to choose the appropriate pain scale (Faces Pain Scale [FPS] [7]; Visual Analogue Scale [VAS] [8]; Numeric Rating Scale [NRS] [9]) to measure pain intensity. An experienced pain nurse instructed him in how to use his choice, the FPS. His FPS reports were further converted to the VAS scale (100 indicating maximum discomfort, 0 indicating maximum comfort).

Prior to testing, the patient was asked to report or imagine his average experience of pain, pruritus, burning, fear, and discomfort when resting and during an average wound care experience (Table 2). As a limitation, reports based on recall may cause bias in estimation.

**TABLE 2:** Patient‐reported average baseline variables at rest^a^ and during wound care^b^. Location: Overall. Reported by Faces Pain Scale converted to Visual Analogue Scale (VAS; 100 indicating maximum discomfort, 0 indicating maximum comfort).

Variable	Baseline when resting^a^ (VAS)	Baseline during wound care^b^ (VAS)
Pain	0	50
Itch	0	62
Burn	0	98
Fear	N/A	100
Discomfort	N/A	100

^a^ How much pain/itch/burn are you usually experiencing when resting?

^b^ When you recall your latest wound care, how much pain/itch/burn did you experience? If you think that we would start wound care now, how much fear/discomfort would you experience?

Second, he was asked: *How well do you think you will manage during this day?* On the VAS scale, his response was 100 (maximum discomfort).

**FIGURE 1:**
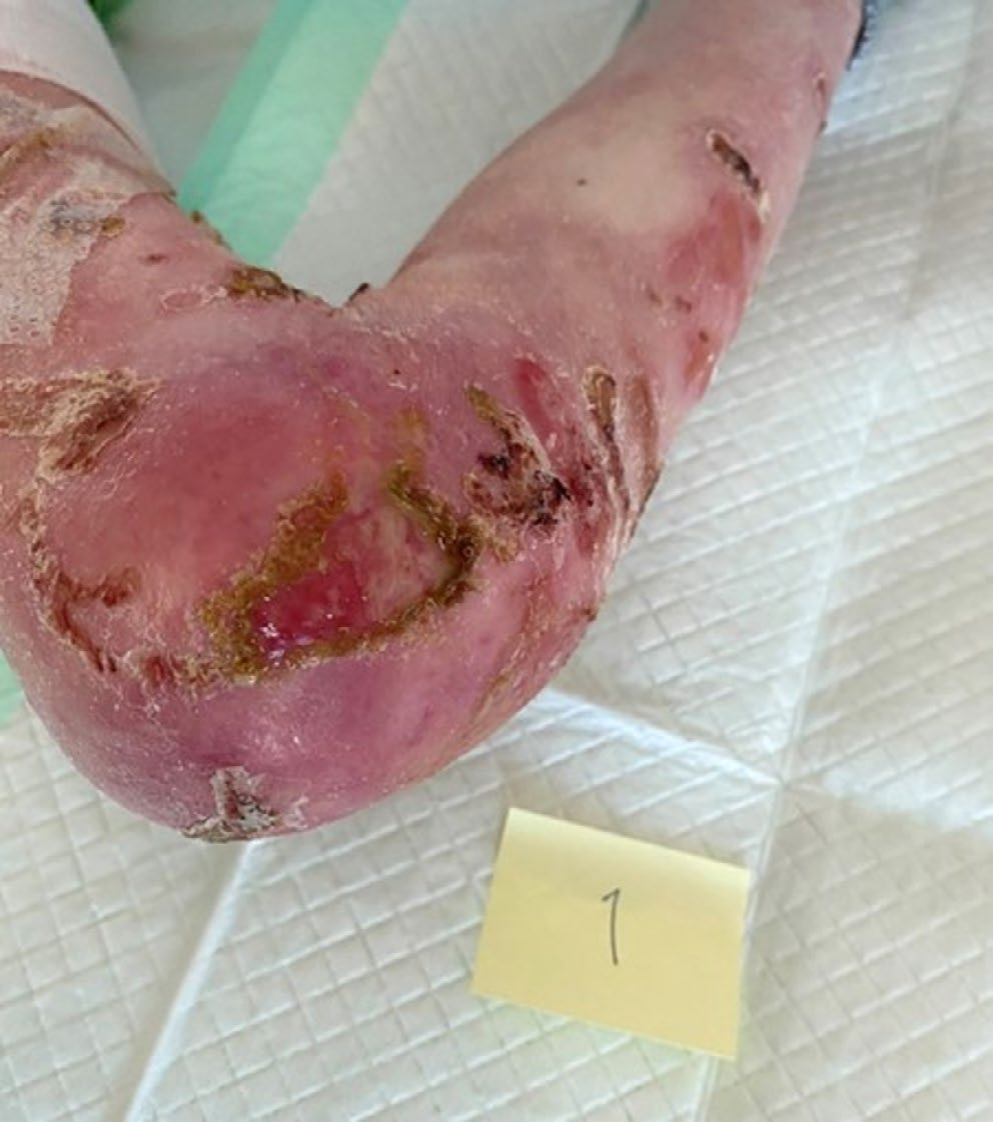
Skin lesion selected for topical product testing: Knee anterior (1).

**FIGURE 2:**
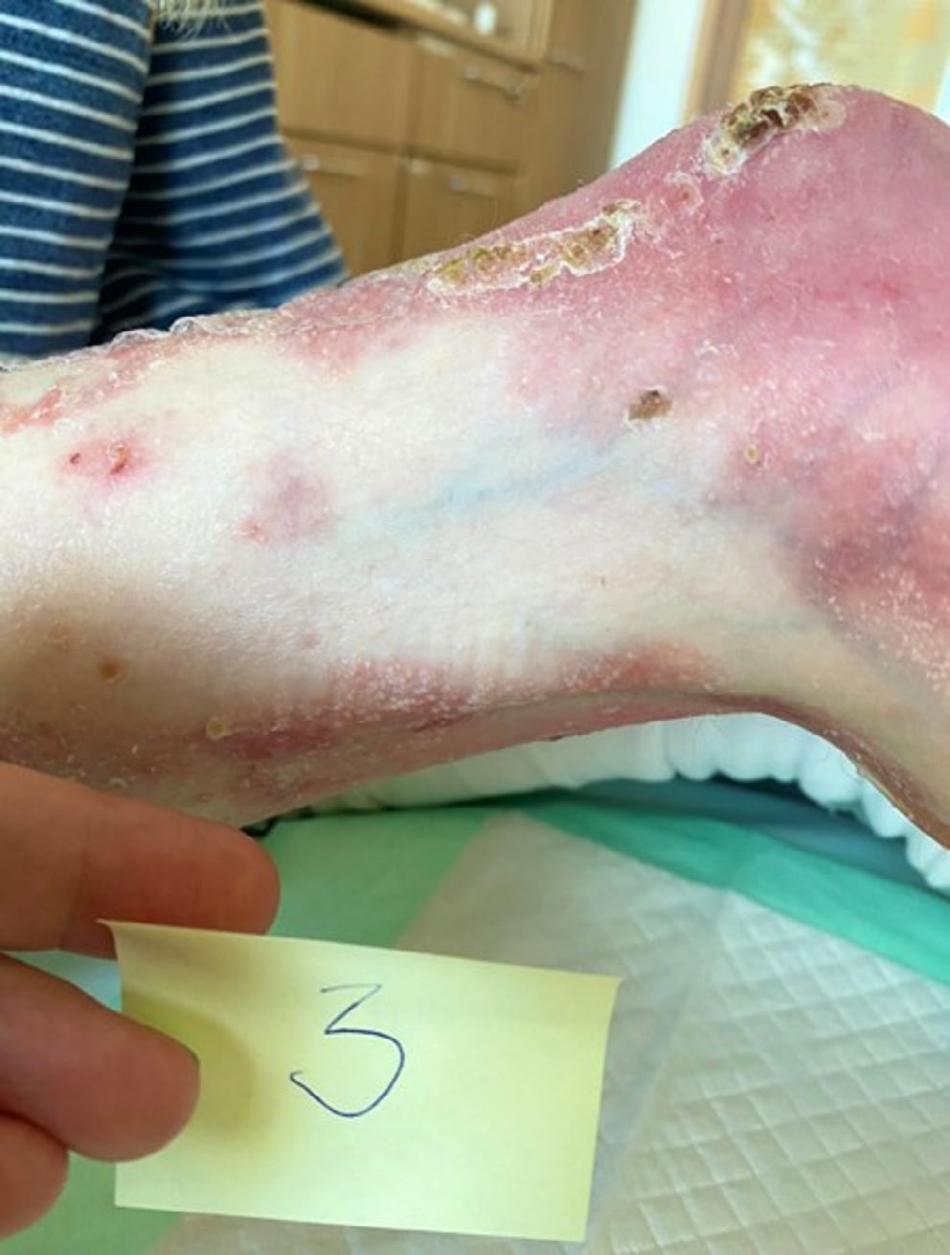
Skin lesion selected for topical product testing: Ham (3).

Four topical products were tested for the clinically most appropriate skin lesion area (Figures 1 and 2). The rationale for selecting these areas was the high patient‐reported symptom intensity and frequency of the selected areas. The patient was asked to report his experience of pain, pruritus, burning, fear and discomfort. (1) immediately before and (2) during topical product administration, and (3) 30 min after topical product administration during wound care (cleaning, wiping, removing crust). Wound care products were not used during or after testing.

Product testing was carried out as planned, and the patient tolerated all products well. Table 3 illustrates the product contents, tested skin areas and lesion types, and the patient reports.

**TABLE 3:** Patient‐reported variables right before and during administration of four topical products and during wound care 30 min after topical product administration. Reported by Faces Pain Scale [9] converted to Visual Analogue Scale [10] (VAS; 100 indicating maximum discomfort, 0 indicating maximum comfort).

Order of testing	Product	Skin area	Variable	Before administration (VAS)	During administration (VAS)	During wound care (VAS)	Notes
1	Lidocaine spray^c^	Knee anterior, Figure 1					
			Pain	76 when flexing the knee or walking	0	0 when wound cared for by self, 30 when wound cared for by physician	
			Itch	0	0	0	
			Burn	0	0	0	
			Fear	100	0*	0 when wound cared for by self; 40 when wound cared for by physician	*“actually it was funny”
			Discomfort	N/A	0	0	
2	Lidocaine gel^d^	Knee lateral, area similar to Figure 1					
			Pain	0	0	40 when wound cared for by self	
			Itch	0	0	0	
			Burn	0	0	0	
			Fear	100	82	N/A	
			Discomfort	N/A	0*	N/A	*“Feels cold”
3	Gabapentin‐lidocaine gel^a^, ^e^, AND gabapentin‐gel^b^, ^f^	Ham, Figure 2					
			Pain	0	0	N/A	
			Itch	40	0*	N/A	*“*All the itching stopped 5 min after administration and I started dancing and jumping”
			Burn	0	0	N/A	
			Fear	100	0	N/A	
			Discomfort	100*	0	N/A	*Tired, wants to go home

^a^ For wounded lesion area.

^b^ For dry area.

^c^ Lidocaine spray: 100 mg/mL.

^d^ Lidocaine gel: 2%.

^e^ Gabapentin‐lidocaine gel: gabapentin 4 g cum lidocaine 2% gel ad 50 g.

^f^ Gabapentin gel: gabapentin 2 g, macrogol 400 2.5 g ad basal crème 45.5 g.

* Notes reflect the child's own reported experiences and comments.

A psychologist from the Pediatric Pain Center met the patient and his mother during the testing day at the ward. The main aim was to enhance patient and maternal self‐efficacy. The importance of recognizing pain‐related fear‐avoidance behavior and the role of pain expectation in pain experience was also discussed.

At the end of the testing day, the patient was asked: *How well do you think you managed during this day?* His response was 0 on the VAS scale (maximum comfort).

## 4 Outcome and Follow‐Up

The patient was advised to continue daily pregabalin (1.7 mg/kg × 2 per os). Regarding topical products, he was advised to apply a thin layer of gabapentin gel to itchy skin areas twice per day. Lidocaine gel or spray was advised to be used directly on wet wounds or granulating lesions before wound care. A psychologist's appointment was offered when needed.

Three months after the testing day, the patient's mother described the pain situation as excellent. They had continued the gabapentin gel for itchy skin areas with “very good efficacy and no noticed adverse effects.*”* Lidocaine spray had caused stinging and had been discontinued. However, lidocaine gel had been “efficient and good with no noticed adverse effects” before wound care and had been used occasionally.

Additionally, the mother reported: “It seems that the testing day itself and the following good experience in wound care have resulted in an extreme change in his [the patient's] attitude towards wound care. He has even been willing to shower and stay there for a long time.”

Six months after the testing day, the patient had only one skin infection requiring oral antibiotic treatment. The patient had been able and motivated to execute wound care himself and had not refused any skin care. Pregabalin was later discontinued due to the improved situation.

## 5 Discussion

Regarding pharmacological management of persistent pain and pruritus in EB, the guideline published in 2014 could not recommend one drug over another [10]. Recently, the effectiveness of topical low‐dose calcipotriol ointment has been suggested [11]. The importance of a non‐pharmacological therapeutic approach has been highlighted [10, 12].

Importantly, to date no studies exist considering either pain management in patients with RDEB‐*SG* specifically or pain management during regular wound care in RDEB. One case report has reported the effectiveness of oral purified cannabidiol in RDEB but only as a palliative approach [13]. Future studies are urgently needed, as RDEB‐*SG* causes extreme suffering for affected children and their caregivers.

Considering the patient presented herein, several matters needed to be taken into account in a comprehensive pain management plan. IV analgesics or drugs needing monitoring were not considerable due to the need for daily and at‐home administration. Opioids and nonsteroidal anti‐inflammatory drugs were not considered due to adverse effects in long‐term use. Further, tricyclic antidepressants should be used with caution due to their potential to prolong QT‐interval in RDEB patients because they carry an increased risk for cardiomyopathy [14]. Topical products were thus preferred due to their relatively good efficacy‐adverse effect ratio.

The difficult combination of frequent primary and treatment‐induced pain, physical restrictions, and living with a rare, suffering‐provoking disease with uncertain prognosis predisposes children to multiple psychological problems, especially when they enter school and when they reach their teen years [15, 16]. Regarding the current patient, it seems that the positive experience regarding wound care during the testing day and, further, improved self‐efficacy played a central role in the patient's improved condition along with optimized pharmacological symptom management. Overall, the management of patients with RDEB‐SG requires a multidisciplinary approach involving dermatology, pain medicine, nursing, psychology, rehabilitation services, and dental care.

This report has several limitations. First, the observations are based on a single patient and therefore cannot be generalized to all patients with RDEB‐SG. Second, no validated measures of health‐related quality of life or pain‐related disability were collected. Third, the outcomes relied largely on patient‐ and caregiver‐reported experiences. Finally, because pregabalin, topical treatments, and psychological interventions were combined as part of a multimodal treatment approach, the relative contribution of each intervention to the observed improvement cannot be determined.

## Author Contributions

**Maiju K. Marttinen:** conceptualization, data curation, investigation, methodology, project administration, writing – original draft, writing – review and editing. **Reetta Sipilä:** conceptualization, investigation, methodology, writing – review and editing. **Hanna Vuorimaa:** conceptualization, methodology, writing – review and editing. **Minna Ståhl:** conceptualization, writing – review and editing. **Katariina Hannula‐Jouppi:** conceptualization, project administration, writing – review and editing.

## Funding

The authors have nothing to report.

## Consent

Both the patient and his caregiver gave their written informed consent for the patient's medical details to be published as a case report.

## Conflicts of Interest

The authors declare no conflicts of interest.

## Data Availability

The data supporting the findings of this study are not publicly available due to patient privacy and ethical restrictions. Additional information may be available from the corresponding author upon reasonable request, subject to applicable ethical and legal requirements.
